# The experiences and perceptions of mental health service provision at a primary health centre in the Eastern Cape

**DOI:** 10.4102/sajpsychiatry.v27i0.1641

**Published:** 2021-08-13

**Authors:** Duane Booysen, Phumeza Mahe-Poyo, Rosemary Grant

**Affiliations:** 1 Department of Psychology, Faculty of Humanities, Rhodes University, Grahamstown, South Africa; 2 Private Consultant, Grahamstown, South Africa

**Keywords:** mental disorder, patients experience and perceptions, primary healthcare, service provision, qualitative, low research setting

## Abstract

**Background:**

Since 1994, the South African healthcare system has undergone several changes to meet the needs of contemporary South Africa. Yet the state of mental healthcare, especially in low-resource areas, remains in a precarious state.

**Aim:**

This study aimed to explore how persons diagnosed with a mental disorder experience and perceive mental health services in a low-resource community in the Eastern Cape, South Africa.

**Setting:**

The study was conducted at a primary care clinic in a low resource community setting in the Eastern Cape, South Africa.

**Method:**

Semi-structured interviews were conducted with eight participants diagnosed with mental illness who had been accessing treatment for at least the past 6 months from a primary health clinic. Thematic analysis was used to analyse and identify pertinent themes.

**Results:**

The following themes emerged from the data: (1) perceptions of mental disorders – role of culture, (2) experiences of having mental disorders – loss of employment, (3) problem of stigma – social rejection and labelling, (4) experience of distress – sadness and frustration and (5) challenges in accessing treatment – transport fee and shortage of staff.

**Conclusion:**

This study yielded several lived experiences and perceptions in relation to participants’ feelings, opinions and interpretations of persons living with mental disorders and accessing mental health treatment in their local context. Future interventions should consider provision of more extensive professional help in the form of counsellors and social workers at the clinics, more efficient service delivery and future interventions regarding stigma should incorporate community members into the learning process.

## Introduction

The provision of mental healthcare in sub-Saharan African countries remains sparse, whilst the impact of mental and substance use disorders continues to add to the burden of disease in African countries.^1^ In the case of South Africa, and lower resourced provinces such as the Eastern Cape, the provision of mental health services are still limited by various factors such as socio-economics status, early detection of mental disorders and limited access to mental healthcare in low-resource communities.^2^ Considering the persistent issues of financial and systemic limitation, exploring the nature and experience of mental health service provision in low-resource communities remain to an important indicator to assess the state of mental health service provision.^3^

Studies by Monteiro^3^ indicated that 30% of the global population suffer from mental disorders, amongst whom are those who do not receive adequate treatment. Gyamfi et al.^4^ indicated that according to World Mental Health Surveys carried out in the America, Europe, Middle East, Africa and Asia, up to 40% of individuals in developed countries and 80% in less developed countries receive no treatment.^4^ To illustrate this, a significant section (75%) of the South African population living with mental disorders are not receiving mental health services.^5^ These findings lead to mental illness being recognised as a significant cause of one of the comorbid illnesses leading to ill health and poor quality of life.

The state of mental health services remains a public health and human rights concern. Yet those who do engage in the available services still encounter various challenges, namely limited resource,^6^ stigma,^7^ social isolation^8^ and being exposed to distressing and harmful experiences when accessing services regarding mental health,^9^ particularly in developing countries such as South Africa.

In addition, mental health services are not prioritised in South Africa.^6^ This is evident by the high incidence of mental disorders and the significant contribution to the burden of disease in South Africa.^5^ This means that approximately one in six South Africans is likely to experience a mental disorder, such as depression, anxiety or substance use, during a typical year.^5^ Thus, mental illness contributes 42% to the burden of disease in South Africa.^2^ Women have the highest incidence rate of mental illness and those living in low- and middle-income areas are most vulnerable to experiencing mental illness.^10^ De Kock et al.^11^ stated that one in 10 participants in a study conducted in these low- and middle-income areas experienced mental disorders.

This situation is further evidenced by the tragic deaths of more than 140 mentally ill patients under the patient care management of the Gauteng Department of Health (GDoH) in 2018.^12^ The loss of life resulted from the rushed execution of the Gauteng Mental Health Marathon Project, when the GDoH ‘precipitously’ terminated its contract with Life Esidimeni, a facility that provided ‘highly specialised chronic psychiatric care’ (p. 1) to mentally ill patients on behalf of GDoH.^12^ As a result, the shortage of mental health staff, coupled with inadequate mental health services, reduces the quality of life amongst persons requiring treatment in South Africa. For example, in 2018 the state of psychiatric hospitals in the Eastern Cape, such as Tower Hospital in Fort Beaufort, concerningly had only one psychiatrist whilst it serves a large portion of the entire province of the Eastern Cape, with an estimated population of 6.9 million, having only 400 beds for 344 males and 56 females.^1^

Considering the precarious state of mental healthcare in South Africa and with the recent findings of the South African Human Rights Commission (SAHRC)^6^ report, the commission found several shortcomings, namely a lack of policy implementation, underinvestment in mental health by government and access to mental health is still focussed at a tertiary level and not at primary care level, amongst others.^6^ Considering the precarious state of mental healthcare in South Africa, it was deemed necessary to explore the experiences and perceptions of mental health service provision at a primary care level in the Eastern Cape.

## Research methods and design

The study employed a descriptive qualitative research method using semi-structured interviews.^13^ The study sample consisted of eight (*n* = 8) participants diagnosed with a mental disorder and who had accessed mental healthcare for at least 6 months at a primary health clinic in the Eastern Cape. Participants’ ages were between 40 and 60 years (*M* = 50 years) and all had received treatment for schizophrenia or major depressive disorder for more than 6 months from a primary healthcare clinic in the Eastern Cape (see Table 1). Purposive sampling was utilised to ensure that participants were receiving treatment for a known mental illness or had experience of living with a mental disorder and accessing treatment at a primary healthcare level.^14^

**TABLE 1:** Participant demographics.

Participants	Gender	Age	Diagnosis	Treatment	Education	Living	Marital status	Income
1	Female	60	Schizophrenia	2003	None	Self	Single	Social grant
2	Male	48	Schizophrenia	1987	Primary	Family	Single	Disability grant
3	Female	43	Depression	2015	Secondary	Family	Single	Disability grant
4	Male	42	Schizophrenia	1998	Secondary	Family	Single	Unemployed
5	Female	47	Depression	2004	Primary	Family	Married	Disability grant
6	Male	43	Schizophrenia	2001	Primary	Single	Single	Casual jobs
7	Male	57	Schizophrenia	2013	Primary	Family	Separated	Disability grant
8	Female	60	Schizophrenia	2019	Primary	Family	Widowed	Social grant

### Procedure

After ethical clearance was granted, the second author, P.M., met with the manager of the identified primary healthcare clinic to discuss and initiate the recruitment process. During the recruitment phase, P.M. was introduced to potential participants who were waiting for services at the clinic and those who indicated interest were invited for a detailed briefing in a private consultation room at the clinic. Participants attended a briefing session with P.M. about the study, which was performed in isiXhosa and in English. Participants were assured that their anonymity and confidentiality would be maintained throughout their participation and that they would have the right to withdraw from the study at any point during the research. Questions and or concerns were addressed to ensure that the possible participants were adequately informed about the nature of the study. At the end of this recruitment sessions, P.M. collected recruitment documents, which included consent forms. Ultimately, there were nine (*N* = 9) willing candidates, with one being unable to provide comprehensive feedback as he did not understand the questions and was deemed inappropriate to be included as a participant in the study. Participant psychiatric history (i.e. diagnosis) was confirmed by the clinic manager of the research site.

### Data collection

All semi-structured interviews were conducted in isiXhosa and recorded with an audio recording device. Audio recordings were translated from isiXhosa to English and transcribed by P.M. The accuracy of the translated transcripts was checked by P.M. and D.B. Interview questions explored how participants experienced living with a mental disorder and their experience of mental healthcare in their local area (see Appendix 1). Transcripts were translated into English for analysis, primarily for the benefit of critical discussion amongst the authors and to disseminate the research findings. The length of interviews was between 40 min and 60 min.

### Data analysis

Data analysis was guided by a thematic analysis,^15^ which is open, flexible and can potentially generate new insights in order to enrich understanding of the way people construct their everyday experiences and perceptions regarding mental health services.^13^ Familiarising ourselves with the participant data through transcribing, reading, and rereading the transcriptions, noting down initial ideas and generating initial codes by coding interesting features of the data in a systematic fashion across the entire data set, collating data relevant to each code and searching for themes by collating codes into potential themes and gathering all data relevant to each potential theme.^15,16^ Lastly, data analysis and writing of the results had undergone an iterative process with the authors discussing and reflecting on the analysis of the data.

### Ethical considerations

The study obtained ethical approval from the Rhodes University Ethical Standards Committee and gatekeeper permission was also obtained from the Eastern Cape Health Research Committee (Reference number: EC_201906_004).

## Results and discussion

A summary of the themes and sub-themes that were identified in the analysis is presented (Table 2).

**TABLE 2:** List of super-ordinate and sub-themes.

Super-ordinate theme	Sub-theme
Perceptions of mental disorders	Role of culture
Experiences of having disorders	Loss of employment
The problem of stigma	Social rejection and labelling
Experiences of distress	Sadness and frustration
Challenges in accessing treatment	Transport fee and shortage of staff

### Theme 1: Perceptions of mental disorders: Role of culture

Bakow and Low^17^ suggested that the role of culture in mental health is not well understood in spite of the fact that the cultural conception of the self has a powerful influence on the manner in which the disorder is expressed and understood. Similarly, in this study, the majority of participants’ understanding of mental disorder is informed by an indigenous cultural perspective. This sub-theme thus highlights the manner in which participants perceive mental disorder and how it influences their cultural beliefs. This is highlighted here:

‘I was taken to a traditional healer who described witchcraft as the cause of my illness …; I also went to the hospital; the blood tests were done but the results came clean; I think it is … because I have been … bewitched.’ (Participant 3, female, 43 years old)

From the given extract, it is clear that following consultation with the traditional healer, the participant sought confirmation from the medical professional. This exemplifies that participants made sense of their illness from both cultural perspective and social context in which they found themselves. It is evident that understanding mental disorder in terms of issues such as witchcraft, compared with being diagnosed medically, requires a complete paradigm shift, one which is difficult, especially for individuals who have not been taught concepts relating to Western medicine. This finding presents as the cultural component that might implicate mental health, as described in the study by Gopalkrishnan,^18^ who described culture as a multilayered concept that is influenced by a range of issues through a set of values and norms followed and created by people.

The role of culture produced insightful discussion during the interview regarding issues of worshiping of the ancestors, which is described as the path to healing and viewed as vital for the maintenance of health within traditional African communities.^17^ The following participants demonstrate how culture influences the manner in which mental disorder is expressed and understood:

‘I think my sickness is caused by the ritual that I needed to be performed, so that I can be protected and my things to go well.’ (Participant 6, male, 43 years old)

‘I believe it’s amafufunyana … people are jealous.’ (Participant 8, female, 60 years old)

The given excerpts imply that participants have a particular understanding of mental disorders and that culture plays a key role in their conceptualisation. Furthermore, it is evident that cultural beliefs play an important role in decision-making regarding assessment and treatment of mental disorders. The majority of participants believe that mental disorders are prompted by witchcraft, and are intentionally caused, for example, as a result of jealousy, someone casts a ‘demon’ on the mentally ill person. As a result of these strong beliefs, the participants sought reassurance from traditional healers and some seem to be suggesting that they had become mentally ill because they had not undergone the necessary rituals, which would ensure that they obtain protection from their ancestors. This is supported by the South African study conducted by Tilolo et al.^19^ who suggested that persons with mental illness believe that mental illness is caused by ancestral anger at their failure to perform rituals. Witchcraft thus played a role in the development of their illness.^20^

### Theme 2: Experiences of having mental disorders – Loss of employment

It is significant to note that unemployment is still a major challenge for people living with mental disorders. Moreover, most of the participants in this study reported being at risk of losing employment after the onset of their illness. A possible reason for this might be that the South African system excludes or denies people with mental disorders a right to work because it lacks reasonable accommodation or is unable to transition to their needs.^6^ Furthermore, the study by Folb et al.^21^ highlighted that receipt of psychiatric treatment is more common amongst those in disadvantaged socioeconomic positions, who are unemployed, and have no income. Its evidence can be found in the following statements:

‘I was working in George … after getting sick I lost the job.’ (Participant 2, male, 48 years old)

Similarly, this view was expressed by Participant 4:

‘I lost many jobs and had to be returned home.’ (Participant 4, male, 42 years old)

It would seem from the given excerpts that loss of employment is experienced by participants living with mental disorder. Furthermore, it appears that unemployment might be viewed as a burden that individuals experience during periods in which they are affected by mental disorder. Folb et al.^21^ demonstrated that people from socially disadvantaged backgrounds are most likely to be unemployed.

### Theme 3: The problem of stigma – Social rejection and labelling

Participants described stigma as a painful and distressing experience and a significant barrier to the inclusion of persons living with mental disorders in community activities, healthcare service, workplaces and accessing education. All participants described the challenges they experience as persons living with mental disorders for more than 6 months and residing in resource-constrained areas.

Most of the participants in this study reported a level of social rejection and stigmatisation resulting in their isolation from the community. The following statements were made by the participants:

‘People chase me away, no one wants to be around me.’ (Participant 7, male, 57 years old)

Similarly, at the time of the interview, Participant 4 was experiencing social rejection and he reported that:

‘People do not want to be associated with me.’ (Participant 4, male, 42 years old)

Based on the above-mentioned excerpts, it is evident that most of the participants in this study were stigmatised and rejected. As a result, it is argued that participants had limited recovery capital, which was a significant obstacle to their mental healthcare improvement.

Research by Eksteen et al.^7^ suggested that most persons living with mental illness are likely to be labelled by the communities and, moreover, at times, they label themselves, which only serves to increase these individuals’ sense of being considered different and possibly even deviant. These results were found to concur with the findings of this study. This is evident in the following excerpt:

‘[*P*]eople always call me names such as crazy and un-progressive person.’ (Participant 4, male, 42 years old)

Participant 5 observed that:

‘People are most likely to describe me as a crazy woman who is unable to look after her children.’ (Participant 5, female, 47 years old)

Similarly, Participant 2 noticed that:

‘People call me a crazy person.’ (Participant 2, male, 48 years old)

The above excerpts by Participant 4 (male, 42 years old), Participant 5 (male, 47 years old) and Participant 2 (male, 48 years old) imply that people with mental disorder experience a sense of being labelled because of the negative way in which society views mental disorders.^22^ Furthermore, a study by Semrau et al.^23^ indicated that an estimated rate of 85% of the world population experiences a sense of being labelled and excluded from participating in a number of different areas of society.^22^

### Theme 4: Experience of distress – Sadness and frustration

The following theme describes the emotional experiences of the participants and how living with a mental disorder resulted in poignant emotional experiences. Participants were asked to describe their experience of mental illness, they expressed a range of negative emotions, including sadness and feeling hurt; denial or non-acceptance of their illness; a feeling of failure and poor self-worth and a feeling of frustration and being ‘crazy’.

The participants felt that it was important to explain their experience of mental disorders, which is accompanied by a deep sense of sadness or hurt. The study by Burns^24^ found lack of understanding mental health-related difficulties that lead to feelings of sadness and hopelessness. A possible reason for this might be that mental disorder is recognised as a significant cause of hurtful experience, as highlighted by Kabir, Iliyasu, Abubakar and Aliyu^25^ and Mayers et al.^9^ Most of the participants’ experiences are explained here. For example, Participant 1 (female, 60 years old) stated that she ‘was sad’ because she ‘was not born like this’.

Meanwhile, Participant 7 expressed sadness because she:

‘[*D*]id not know what stress is and did not know what caused it.’ (Participant 7, male, 57 years old)

Similarly, Participant 8 described how she:

‘[*F*]elt sad … Because she did not really understand what she was suffering from.’ (Participant 8, female, 60 years old)

This emotion was reinforced by Participant 3 and Participant 5 who both expressed feeling hurt because they perceived themselves to be a burden on their families:

‘I feel hurt because I feel like a burden to my mother who is always looking after me.’ (Participant 3, female, 43 years old)

‘I feel hurt because I cannot take the mother’s role to my children.’ (Participant 5, female, 47 years old)

It seems that a lack of knowledge was an important aspect of the feeling of sadness experienced by the participants. It is this lack of understanding or ‘not knowing’ which seemed to exacerbate the feelings of helplessness, which accompanied the mental illness.

Furthermore, most participants in this study reported frustration at not being able to do the things the patient was able to do prior to suffering from her diagnosis. The following excerpt explains:

‘I first felt frustrated because I lost my job, even if I have a job offer my employer does not trust me.’ (Participant 4, male, 42 years old)

Whilst Participant 1 (female, 60 years old) expressed frustration ‘because there are so many things I can’t do’, other participants, such as Participant 5 (female, 47 years old) stated that ‘there are times I feel like I am crazy’.

It is clear from the interviews that a mental disorder was accompanied by a broad range of feelings, including that of frustration at not being able to do the things the patient was able to do prior to suffering from a mental illness. Whilst it is not possible to interpret exactly what feeling ‘crazy’ means, it clearly leaves a person feeling confused and distressed.

### Theme 5: Challenges in accessing treatment – Transport fee and shortage of staff

A study by Vergunst^26^ indicated that rural communities in South Africa experience significant barriers to accessing healthcare. It is important to consider this relatively recent research publication at this juncture, as it demonstrates about the current the challenges of persons living with mental disorders. The study by Vergunst^26^ highlighted that the high cost of transport and the long distances needed to be travelled makes it increasingly burdensome for people from the rural areas to access healthcare. This problem was expressed by the following participant in this study:

‘[*I*]f there is no medication on the date given then you have to go to the hospital, which means I have to go home and borrow some taxi money because the hospital is far from where I stay.’ (Participant 1, female, 60 years old)

The experience of Participant 1 is supported by the SAHRC^6^ which highlighted that the Rural Mental Health Campaign conducted in 2015 noticed that medication stock-outs are a significant challenge in rural parts of the country and a very limited number of different types of psychotropic medicines are available from National Department of Health.

Furthermore, most participants in the study described shortages of staff as a significant challenge within the facilities where they access their treatment, despite the treatment they receive. This is supported by a study by Vergunst^26^ who suggested that rural areas in South Africa often do not have psychiatrists or psychologists and rely primarily on general practitioners (GPs) and nurses for mental health interventions. This has been highlighted further by a study by the SAHRC^6^ which indicated that in 2017 the South African Society of Psychiatry reported that there were only six psychiatrists working in the entire public mental health system in the Limpopo province and facilities such as Hayani specialist mental health hospital did not have a psychiatrist on staff. In his study, the following statements exemplify similar service constraints:

‘I spend many hours in the waiting room before I get treatment.’ (Participant 5, female, 47 years old)

‘[*F*]ew nurses and no one you can speak to.’ (Participant 5, female, 47 years old)

The above-mentioned excerpts signify that there is still a shortage of mental healthcare staff in the South African healthcare system. These excerpts are supported by the SAHRC^6^, which highlights that the number of psychiatrists and psychologists are low compared with the number of clients in need of care.

## Conclusion

In analysing the textual data of patients diagnosed with mental disorders and using a socially constructionist informed thematic analysis framework, several findings reflected the experiences and perceptions of these patients in relation to their feelings, opinions and interpretations of living with mental disorders and accessing mental health treatment in their local context. This study found how the participants understand and make sense of mental disorders in the interview context considering the research relationship, social, historical and cultural elements that further influence their perceptions, decisions and actions that might have an impact on their well-being.

Participants expressed practical distress complicating some of their relationships and highlighted lack of knowledge, education, stigma and lack of efficiency in terms of service delivery. As a result, participants tended to position themselves as responsible for their inability to function at optimal levels within the social world. Furthermore, this study highlights the experience of social withdrawal, social judgment and labelling of people living with mental disorders, which was fuelled by a lack of knowledge, support, shortage of staff and minimal education about mental disorders, not only amongst the patients but also within their communities.

### Limitations of the study

A limitation of this study is that the interviews were conducted with people in their home language, namely isiXhosa, which then had to be translated that constricted the essential meaning of what participants were saying. This has been confirmed by a study by Twin^27^ in which it is stated that working with translated text often means that some of the meaning gets lost in translation. Lastly, the study is also limited by sample size and its location. Thus, the findings of the study cannot be generalised to greater context of South Africa.

### Contributions

Despite the above-mentioned limitations, the study has offered the participants an opportunity to talk about their personal experiences and perceptions of their own mental health and the experiences of mental health services in a resource-constrained context. In addition, the study highlighted how persons living with a mental disorder still encounter various challenges related to mental health, and how the South African mental healthcare system at a primary care level remains limited.

### Recommendations

Future interventions should consider provision of more professional help in the form of counsellors and social workers at the clinics, more efficient service delivery and providing education regarding mental disorders, especially for the families of those affected. Furthermore, the study demonstrated that issues of culture have a significant influence on the manner in which patients understand their experiences. Moreover, future interventions regarding stigma should incorporate community members into the learning process.

It is clear from this and other research that lack of resources^28^, shortage of mental health staff, coupled with inadequate mental health services reduce the quality of life amongst persons requiring treatment in South Africa.^29^ In line with previous findings by Vergunst,^26^ this study suggests that in order to improve the management of mental illness in rural areas, increased resources need to be made available to improve the quality of treatment.

## Appendix 1

**TABLE 1-A1:** Interview schedule.

Number	Question
1.	How did you find out about your illness? When did you first find out? What was it like when you first heard the news? Did you understand the illness?
2.	Can you tell me about your experiences and perception of having mental illness? How did you feel about it? How did you cope?
3.	Are there any mental health institutions in your area? Yes or No (Circle) If yes, name them.
4.	Which mental health services are being rendered? Which services would you like to be rendered?
5.	Have you used them? Yes or No (Circle) If no, what are the reasons for not using the services? If yes, what kind of service did you receive? How was the treatment by the mental health workers?
6.	How would you describe living with mental illness? What are the positives? What are the negatives?
7.	How would you describe accessing services for mental illness in your area? Are there any stigmas about mental health in your area?
8.	Suggestions to improve mental services: What do you recommend should be performed regarding the improvement of mental services in your area? What other suggestions do you have for making services better?

## References

[1] Sukeri K. Regional aspects of long-term public sector psychiatric care in the Eastern Cape. S Afr J Psychiatr. 2017;23(1):a992. 10.4102/sajpsychiatry.v23i0.992 PMC6138187 30263179

[2] Lund C, Alem A, Schneider M, et al. Generating evidence to narrow the treatment gap for mental disorders in sub-Saharan Africa: Rationale, overview and methods of AFFIRM. Epidemiol Psychiatr Sci. 2015;24(3):233–240. 10.1017/S2045796015000281 25833714 PMC4491538

[3] Monteiro NM. Addressing mental illness in Africa: Global health challenges and local opportunities. Community Psychol Glob Perspect. 2015;1(2):78–95. 10.1285/i24212113v1i2p78

[4] Gyamfi S, Hegadoren K, Park T. Individual factors that influence experiences and perceptions of stigma and discrimination towards people with mental illness in Ghana. Int J Ment Health Nurs. 2018;27(1):368–377. 10.1111/inm.12331 28345310

[5] Lund C, Petersen I, Kleintjes S, et al. Mental health services in South Africa: Taking stock. Afr J Psychiatry. 2012;15(6):402–405. 10.4314/ajpsy.v15i6.48 23160613

[6] South African Human Rights Commission. Report of the National investigative hearing into the status of mental health care in South Africa. Johannesburg: SAHRC; 2019.

[7] Eksteen HC, Becker PJ, Lippi G. Stigmatization towards the mentally ill: Perceptions of psychiatrists, pre-clinical and post-clinical rotation medical students. Int J Soc Psychiatry. 2017;63(8):782–791. 10.1177/0020764017735865 29067838

[8] Sibeko G, Milligan PD, Temmingh H, et al. Caregiving for mental health service users: A study exploring the perceptions of mental health service users and their caregivers in Cape Town, South Africa. Int J Soc Psychiatry. 2016;62(6):512–521. 10.1177/0020764016651458 27282176

[9] Mayers P, Keet N, Winkler G, et al. Mental health service users’ perceptions and experiences of sedation, seclusion and restraint. Int J Soc Psychiatry. 2010;56(1):60–73. 10.1177/0020764008098293 20053723

[10] Rochat TJ, Tomlinson M, Bärnighausen T, et al. The prevalence and clinical presentation of antenatal depression in rural South Africa. J Affect Disord. 2011;135(1–3):362–373. 10.1016/j.jad.2011.08.011 21880372 PMC3210898

[11] De Kock JH, Pillay BJ. A situation analysis of psychiatrists in South Africa’s rural primary healthcare settings. Afr J Prim Health Care Fam Med. 2017;9(1):1–6. 10.4102/phcfm.v9i1.1335 PMC5458564 28582989

[12] Ferlito BA, Dhai A. The life Esidimeni tragedy: The courts are also to blame. S Afr Med J. 2018;108(3):155–156. 10.7196/SAMJ.2018.v108i3.13011 30004354

[13] Terre Blanche M, Kelly K, Durrheim K. Why qualitative research. In: Blanche MT, Blanche MJT, Durrheim K, Painter D, editors. Research in practice: Applied methods for the social sciences. Cape Town: UCT Press, 2006; p. 271–284.

[14] Palinkas LA, Horwitz SM, Green CA, et al. Purposeful sampling for qualitative data collection and analysis in mixed method implementation research. Adm Policy Ment Health. 2015;42(5):533–544. 10.1007/s10488-013-0528-y 24193818 PMC4012002

[15] Braun V, Clarke V. Using thematic analysis in psychology. Qual Res Psychol. 2006;3(2):77–101. 10.1191/1478088706qp063oa

[16] Parker I. Social constructionism, discourse and realism. London: Sage; 2005.

[17] Bakow BR, Low K. A South African experience: Cultural determinants of ukuthwasa. J Cross-Cult Psychol. 2018;49(3):436–452. 10.1177/0022022117753546

[18] Gopalkrishnan N. Cultural diversity and mental health: Considerations for policy and practice. Front Public Health. 2018;6:179. 10.3389/fpubh.2018.00179 29971226 PMC6018386

[19] Tilolo L, Magadla NIN, Tshotsho N. Perceptions of indigenous people regarding mental illness at Cacadu district in the Eastern Cape province, South Africa. Afr J Phys Health Educ Recreat Dance. 2015;21(2.2):397–406.

[20] Mbanga NI, Niehaus DJH, Mzamo NC, et al. Attitudes towards and beliefs about schizophrenia in Xhosa families with affected probands. Curationis. 2002;25(1):65–73. 10.4102/curationis.v25i1.718 12096574

[21] Folb N, Lund C, Fairall LR, et al. Socioeconomic predictors and consequences of depression among primary care attenders with non-communicable diseases in the Western Cape, South Africa: Cohort study within a randomised trial. BMC Public Health. 2015;15(1):1194. 10.1186/s12889-015-2509-4 PMC4666155 26621252

[22] Picco L, Pang S, Lau YW, et al. Internalized stigma among psychiatric outpatients: Associations with quality of life, functioning, hope and self-esteem. Psychiatry Res. 2016;246:500–506. 10.1016/j.psychres.2016.10.041 27821360

[23] Semrau M, Evans-Lacko S, Koschorke M, et al. Stigma and discrimination related to mental illness in low- and middle-income countries. Epidemiol Psychiatr Sci. 2015;24(5):382–394. 10.1017/S2045796015000359 25937022 PMC8367357

[24] Burns JK. Poverty, inequality and a political economy of mental health. Epidemiol Psychiatr Sci. 2015 4;24(2):107–113. 10.1017/S2045796015000086 25746820 PMC6998107

[25] Kabir M, Iliyasu Z, Abubakar IS, et al. Perception and beliefs about mental illness among adults in Karfi village, northern Nigeria. BMC Int Health Hum Rights. 2004;4(1):3. 10.1186/1472-698X-4-3 15320952 PMC515308

[26] Vergunst R. From global-to-local: Rural mental health in South Africa. Glob Health Action. 2018;11(1):1413916. 10.1080/16549716.2017.1413916 29320947 PMC7012042

[27] Twin S. An exploratory study examining the influence of translation on the validity and reliability of qualitative data in nursing research. J Adv Nurs. 1997;26(2):418–423. 10.1046/j.1365-2648.1997.1997026418.x 9292378

[28] Petersen I, Lund C. Mental health service delivery in South Africa from 2000 to 2010: One step forward, one step back. S Afr Med J. 2011;101(10):751–757. 22272856

[29] Young C. South African counselling psychology at the crossroads: Lessons to be learned from around the world. S Afr J Psychol. 2013;43(4):422–433. 10.1177/0081246313504697

